# A Rapidly Deteriorating Case of Bivalvular Endocarditis in a Hemodialysis Patient: A Case Report

**DOI:** 10.7759/cureus.69530

**Published:** 2024-09-16

**Authors:** Sachin Kumar, Thomas A Elimihele, Adetayo Y Odueke, Sachika Gandhi

**Affiliations:** 1 Internal Medicine, Spartan Health Sciences University School of Medicine, Vieux Fort, LCA; 2 Internal Medicine, Meharry Medical College, Nashville, USA; 3 Internal Medicine, University of Arkansas for Medical Sciences, Little Rock, USA; 4 Surgery, Montefiore Medical Center, Wakefield Campus, Bronx, USA

**Keywords:** end stage renal disease (esrd), heart vegetations, infective endocarditis, methicillin resistant staphylococcus aureus (mrsa), morbidly obese, transesophageal echocardiography (tee), upper extremity thrombosis

## Abstract

Infective endocarditis (IE) is a rare but potentially life-threatening condition characterized by inflammation and infection of the inner lining of the cardiac chambers, native or prosthetic valves, or indwelling cardiac devices. In recent decades, its incidence has increased exponentially in healthcare-associated settings such as hemodialysis (HD). The primary causative agent is typically *Staphylococcus aureus*, followed by streptococci and, in some instances, even fungal infections, although infectious agents do not exclusively cause the condition.

In this case report, we detail the clinical presentation of a 46-year-old morbidly obese male with a medical history notable for hypertension, poorly controlled diabetes, and end-stage renal disease necessitating HD. Upon arrival at the emergency department, he presented following a two-week lapse in dialysis sessions, reporting symptoms of altered mental status and lethargy. Shortly after that, the patient's condition rapidly deteriorated, marked by fever, vomiting, and indications of septic shock. Physical examination revealed signs consistent with meningism, alongside the identification of a clotted radio-cephalic fistula, impeding vascular access essential for HD. Furthermore, severe uremia was evident, attributed to the prolonged absence of dialysis treatment. Concurrently, given the patient's presentation of meningeal signs, we were concerned about a potential diagnosis of meningitis. Our immediate priority was to stabilize the patient's vital signs and address the resolution of potential uremic encephalopathy. Additionally, we prioritized the investigation of possible sources of bacterial infection that could be contributing to septic shock and sudden deterioration.

This case highlights the complex presentation of IE, which necessitated the collaboration of multidisciplinary teams to address the patient's condition. Additionally, emphasis is placed on HD as a major risk factor for IE, with discussion of associated factors such as constant manipulation of skin flora during dialysis, types of vascular access utilized, and the potential for fistula infection to directly or indirectly contribute to IE. Furthermore, we explore the idea of a possible link between meningism or meningitis and IE.

## Introduction

Infective endocarditis (IE) is a rare but potentially life-threatening condition characterized by inflammation and infection of the inner lining of the cardiac chambers, native or prosthetic valves, or indwelling cardiac devices.

There has been an exponential increase in the incidence of IE in healthcare-associated settings such as hemodialysis (HD) in recent decades [1]. Several studies have shown HD to be a significant independent risk factor for IE. One study noted that approximately one-third of IE cases occurred in patients with end-stage renal disease (ESRD), with 73% of infections originating from vascular sites [2]. Another study, analyzing data from dialysis patients in the United States Renal Data System, reported an age-adjusted incidence rate of 17.86, significantly higher than the general population [3]. The primary causative agent is typically *Staphylococcus aureus*, followed by streptococci, and in some instances, even fungal infections, although infectious agents do not exclusively cause the condition [4].

This case describes the complex presentation of IE, necessitating multidisciplinary collaboration. It emphasizes HD as a significant risk factor, highlighting frequent skin flora manipulation and potential fistula infections. Furthermore, it explores a potential link between meningism or meningitis and IE.

## Case presentation

In this case report, we detail the clinical presentation of a 46-year-old morbidly obese male with a medical history notable for hypertension, poorly controlled diabetes, and ESRD necessitating HD. He presented following a two-week lapse in dialysis sessions, reporting symptoms of altered mental status (AMS) and lethargy.

During the initial assessment in the ER, the patient was awake but rambling and ill-looking. His vital signs were significant for a heart rate of 108 beats per minute, respiratory rate of 23-25 cycles per minute, temperature of 99°F, with normal blood pressure and oxygen saturation. Apart from the tachycardia, the rest of the cardiovascular examination revealed a regular heart rhythm with no murmurs or jugular venous distension. Other notable physical examination findings were the absence of a thrill at the site of the right radio-cephalic fistula (suggestive of a clot at the HD access site) and a left below-the-knee amputation. There were no other significant findings at the initial assessment. While in the ER, the patient vomited twice and, on further assessment, was found to have a fever and exhibited neck stiffness. The patient's initial laboratory results revealed leukocytosis with white cell counts of 16,200 (reference range (RR) 4,000-10,800 cells/uL), severe uremia with creatinine levels of 11.18 mg/dL (RR 0.5-0.8 mg/dL) and blood urea nitrogen (BUN) levels of 86 mg/dL (RR 9-23 mg/dL), mildly elevated high sensitivity troponin I was observed with a lab value of 23 ng/L (RR < 20 ng/L). There is also mild lactatemia with lactate levels at 3 mmol/L (RR 0.5-1.6 mmol/L) and minimal hyperkalemia with potassium levels at 5.6 mEq/L (RR 3.5-5.2 mEq/L).

**Figure 1:**
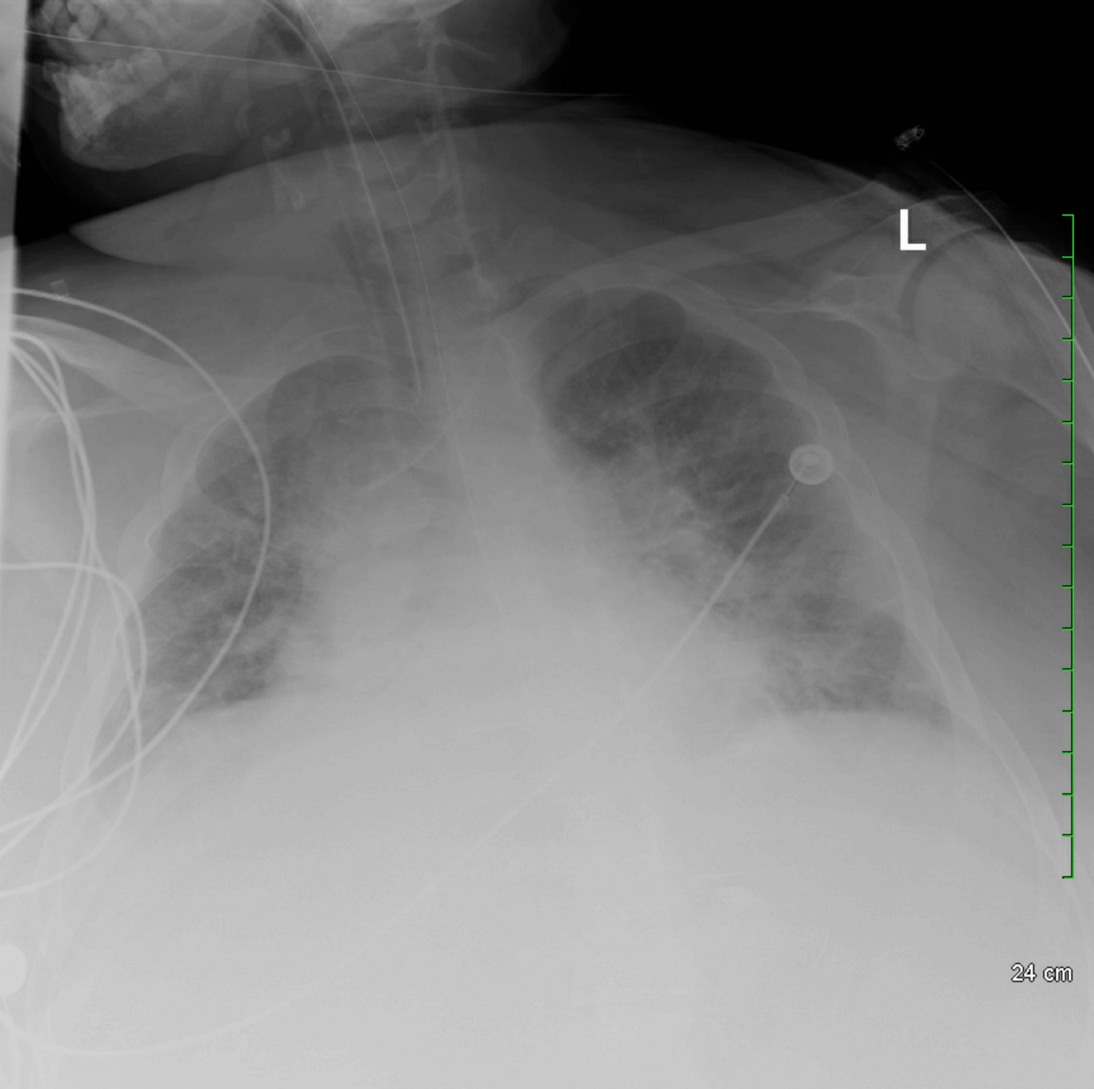
Supine anteroposterior chest view

The X-ray revealed evidence of vascular congestion and poor inspiration (Figure 1), with no improvement in the patient's mental status. Additionally, a head CT scan without contrast showed no evidence of intracranial hemorrhage or evidence of septic embolic stroke. A CT scan of the abdomen without contrast did not reveal any acute changes. 

The patient was given cautious hydration with close monitoring of serum electrolytes, calcium carbonate, and intracellular shifters for mild hyperkalemia, and broad-spectrum antibiotics were initiated to cover possible bacteremia. Nephrology evaluated the patient, who recommended permacath placement - given significantly elevated creatinine levels of 11.18 mg/dL (RR 0.5-0.8 mg/dL) and uremic features supported by the AMS on admission.

Due to continued AMS and the patient becoming hypotensive, a decision was reached to admit them to the ICU. Vascular surgery was consulted and successfully placed a right femoral tunneled HD catheter. The patient was dialyzed with decreased serum creatinine and BUN levels. A transthoracic echocardiogram (TTE) revealed an ejection fraction of 60% with mild concentric hypertrophy. There was suspicion of abnormal mitral valve vegetation; however, poor visualization due to the patient's large body habitus hindered confirmation. Consequently, a transesophageal echocardiogram (TEE) was recommended. Evaluation by infectious disease (ID) showed that the patient was still unresponsive with nuchal rigidity, fever, and leukocytosis. The patient was placed on broad-spectrum antibiotics pending the result of blood culture. From an ID standpoint, the primary concerns were to rule out bacterial meningitis, encephalitis, uremic encephalopathy, and other potential sources of infection. 

On the second day of evaluation, the patient was intubated and maintained on 50% FiO2 to achieve normal oxygen saturation. His blood pressure was 97/45 mm Hg, and his heart rate was 110 beats per minute. Laboratory results showed minimal improvement, with a slight decrease in white cell counts from 16,200 to 15,000 cells/uL (RR 4,000-10,800 cells/uL) and normalization of serum potassium levels at 4.5 mmol/L (RR 3.4-4.5 mmol/L). The creatinine level decreased to 7.9 mg/dL (RR 0.5-0.8 mg/dL) and BUN to 64 mg/dL (RR 9-23 mg/dL). However, his platelet counts dropped from 95,000 to 58,000 units/mcL (RR 150,000-400,000 units/mcL). There was concern for heparin-induced thrombocytopenia (HIT), given that the patient had received heparin for deep vein thrombosis (DVT) prophylaxis. His serum troponin I showed an elevated trend from the day of admission, which was attributed to his ESRD.

**Figure 2:**
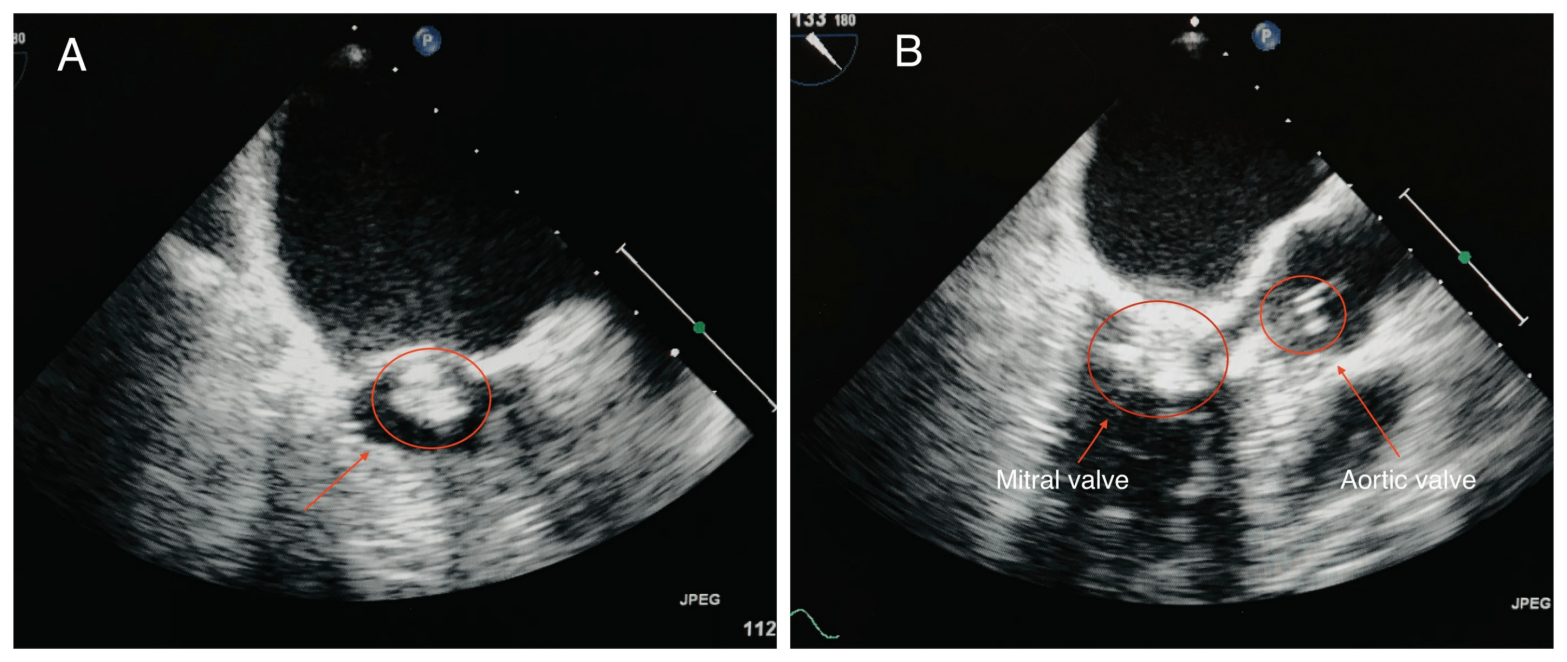
Transesophageal echocardiogram (A) revealing large echodensities attached to the anterior mitral valve leaflets and (B) echodensities on both the mitral and aortic valve leaflets

TEE on day 2 revealed multiple large echo densities on the anterior mitral valve leaflets with mild to moderate regurgitation (Figure 2). Additionally, a large echo density was observed on the ventricular side of the aortic valve, confirming the diagnosis of native bivalvular IE. The blood cultures returned positive for *Staphylococcus aureus*, prompting antibiotic adjustments based on the sensitivity pattern. On a subsequent day, the patient exhibited absent right peripheral pulses in the upper extremity and edema, prompting suspicion of systemic embolization. A stat gray-scale, color duplex Doppler imaging confirmed embolization through evidence of cephalic vein occlusion. Although we had identified the potential source of infection, the patient's deteriorating condition prevented us from performing a lumbar puncture. Consequently, the patient was transferred to a tertiary care center for prompt, specialized treatment and surgical intervention.

## Discussion

IE is a complex and life-threatening condition that sometimes requires multidisciplinary team management, especially when complicated by comorbidities, given its high mortality rate. Most cases of IE in patients on HD occur on the left side of the heart, with *Staphylococcus aureus* being the most common pathogen. Approximately 2-6% of people on HD develop IE, with a 50-60 times higher incidence among dialysis patients compared to the healthy population [5]. A prospective cohort study revealed that both in-hospital and six-month mortality rates were notably higher in HD patients compared to non-HD patients, at 30.4% versus 17% and 39.8% versus 20.7%, respectively, with statistically significant differences (p-value) [6]. Recurrence of IE is also common among HD patients following the initial episode, highlighting the importance of close follow-up to identify recurring cases promptly. The incidence of IE among chronic HD patients is on the rise and often presents with severe morbidity, which can explain the high mortality rate noted above. 

Our case report mirrors this scenario. As presented above, we saw the case of a young man with poor health status who deteriorated quickly shortly after presentation, requiring a higher level of care and even referral due to the complexity of the required care. Therefore, the need for heightened clinical suspicion when these patients present cannot be overemphasized.

In addition to traditional risk factors for IE like structural valve abnormalities and the presence of prosthetic valves, other variables related to the patient's comorbid conditions, like the need and type of vascular access employed, such as arteriovenous (AV) fistulas, temporary catheters, or tunnel catheters further heightens IE risk and severity in these patient population. Furthermore, the duration of HD and the presence of other chronic diseases, such as diabetes mellitus, which can compromise the immune system, exacerbate the already diminished immunity in ESRD [5]. These risk factors are not limited to those mentioned here. According to a study conducted by the Mayo Clinic, individuals utilizing synthetic intravascular dialysis access methods, such as venous catheters and synthetic grafts, demonstrate a higher incidence of IE compared to those with native arteriovenous fistulas [7]. Also, though the association between IE and meningitis is infrequent, it is marked by a high mortality rate and an increased risk of cerebrovascular events, especially in cases involving *Staphylococcus aureus* [8,9]. *Staphylococcus aureus* bacterial meningitis commonly arises from extra-neurological sources, particularly in community-acquired infections [10]. The symptomatic presentation of meningitis often takes precedence, even when it occurs as a complication of IE [9]. Neurological manifestations in patients with negative lumbar puncture results in cases of IE are frequently attributed to sepsis or diminished cardiac output [9]. Despite our efforts, the patient continued to deteriorate, with notable progression of symptoms, including the development of emboli to the arm. Although we had already confirmed the potential source of infection, we were unable to perform a lumbar puncture due to the patient's worsening condition. Consequently, it became necessary to transfer the patient to a tertiary care center for advanced management.

## Conclusions

The clinical manifestations of IE are diverse and frequently ambiguous, underscoring the importance of healthcare providers maintaining a high index of suspicion. Effective care demands a comprehensive approach, integrating early detection, targeted antimicrobial therapy, timely surgical intervention if required, and prompt referral to higher centers as necessary. As IE poses significant clinical hurdles in dialysis settings, ongoing research and vigilant clinical practices are imperative to enhance patient outcomes and reduce mortality risks in this vulnerable population.
